# Idiopathic dilatation of the pulmonary artery: a pediatric case report and literature review

**DOI:** 10.3389/fcvm.2025.1667070

**Published:** 2026-01-08

**Authors:** Jingwei Sun, Jiachen Li, Mengjing Wang, Nan Dong, Dong Qi

**Affiliations:** 1 Pediatrics Department, Bengbu First People’s Hospital, Bengbu, Anhui, China; 2 Ultrasonography Lab, Bengbu First People’s Hospital, Bengbu, Anhui, China; 3 Imaging Department, Bengbu First People’s Hospital, Bengbu, Anhui, China

**Keywords:** cardiovascular abnormalities, case report, idiopathic dilatation of the pulmonary artery, literature review, pediatric

## Abstract

**Background:**

Idiopathic dilatation of the pulmonary artery (IDPA) is a rare vascular anomaly characterized by isolated dilation of the main pulmonary artery. There remains a limited understanding of IDPA, with a limited number of reported cases in the literature.

**Case presentation:**

This study reported a case of a 3-year and 11-month-old female presenting with a 1-month history of cough. The girl was diagnosed with IDPA based on Coronary computed tomography angiography (CCTA) and echocardiography showing significant dilation of the main pulmonary artery (25 mm, *Z*-score: 5.32) and its left (16 mm, *Z*-score: 5.3) and right (17 mm, *Z*-score: 6.08) branches. A systematic analysis of IDPA case reports was then conducted, and a review of relevant literature.

**Conclusion:**

There is a significant gap in available guidance regarding the management and presentation of IDPA. Due to the potential for severe complications, case reports and data collection are critical to advancing IDPA understanding, and ultimately improving management and early identification.

## Introduction

Idiopathic dilatation of the pulmonary artery (IDPA) is a rare vascular anomaly that is specifically characterized by isolated dilation of the main pulmonary artery in the absence of any associated cardiovascular or pulmonary abnormalities, or other identifiable causes (1). It is important to note that while dilation of the pulmonary artery can be observed relatively frequently in the pediatric population, such dilation is often secondary to a range of underlying conditions, including congenital heart disease, pulmonary hypertension, or other structural and functional abnormalities (2). In contrast, the diagnosis of IDPA is established only after exclusion of these known etiologies, rendering it truly idiopathic.

First reported by Wessler and Jaches in 1932, fewer than 100 cases have been documented globally, leaving the underlying pathogenesis and poorly understood etiology (3). Nevertheless, several potential contributing factors have been proposed in the literature. These include congenital defects in elastic tissue development, genetic predisposition, and associations with connective tissue disorders, although definitive causative mechanisms have not been established (1, 3).

Clinical diagnosis is often challenging due to the diverse symptom presentations and clinical mimics. Although IDPA is most commonly diagnosed in adulthood, the presence of pediatric case reports suggests a congenital origin (1). The most distinctive feature of IDPA is the dilation of the main pulmonary artery, which may or may not be accompanied by dilation of its branches (1, 3).

With a low incidence rate of approximately 0.007%, the paucity of available literature highlights the need for updated diagnostic criteria, particularly in light of advancements in imaging modalities, as well as consensus on treatment guidelines (3). Further case reports and comprehensive data collection are crucial to advancing our understanding of IDPA and improving clinical management. Moreover, careful differentiation from secondary causes of pulmonary artery dilation remains essential in both clinical practice and research.

This paper aims to present a case report of IDPA and conduct a systematic analysis and literature review of IDPA cases, as gathering comprehensive data through such reports is crucial for deepening our understanding and advancing clinical knowledge of IDPA.

## Case description

A 3-year and 11-month-old female was admitted to BengBu First People's Hospital with a one-month history of cough, which had gradually worsened over the past two days prior to presentation. Her past medical history included thrombocytopenia and hypersplenism diagnosed at 10 months of age for which she was treated with a short course of oral prednisone tablets. The child has no history of prematurity, and no symptoms of heart-related disease were noted during routine daily life. No cardiac murmurs were detected during previous physical examinations. The abnormally enlarged pulmonary artery was discovered incidentally during routine admission workup when the patient presented for a respiratory tract infection. Further inquiry revealed no family history of inherited heart disease.

On presentation, the patient was conscious and apyrexial (36.5 °C). Her vital signs, including heart rate (120 beats per minute), respiratory rate (26 breaths per minute), and blood pressure (87/58 mmHg), were all within normal limits. Physical examination was otherwise unremarkable, with no observed cardiac murmurs or signs of heart failure. Initial laboratory values showed mild anemia (Hemoglobin: 111 g/L) and moderate thrombocytopenia (Platelets: 72 × 10⁹/L), but were otherwise unremarkable.

**Figure 1:**
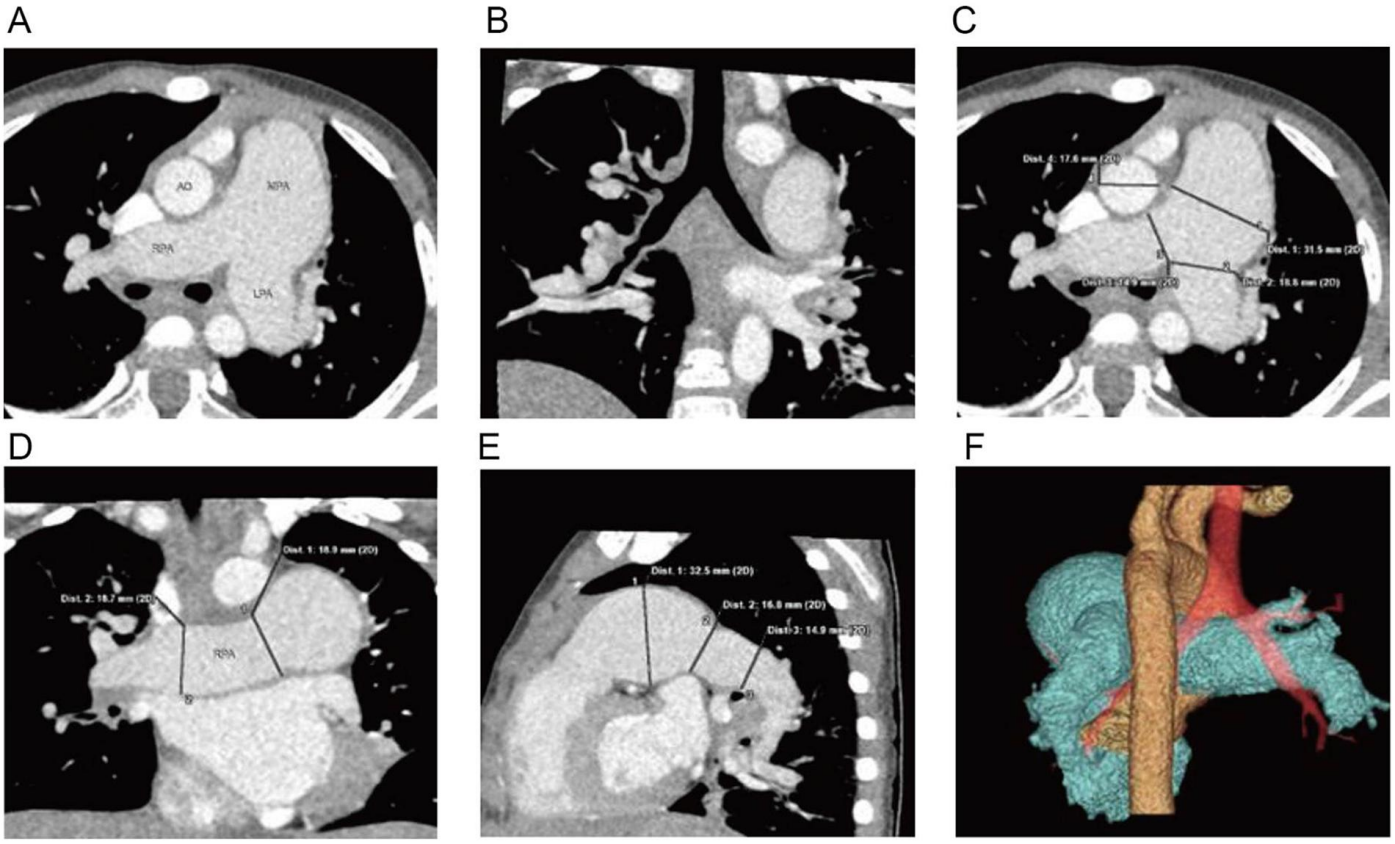
Cardiac CTA: **(A)** significant dilation of the main pulmonary artery and its left and right branches, **(B)** coronal reconstruction reveals mild narrowing of the lumen of the right lower lobe bronchus, **(C–E)** three-dimensional reconstructions displaying the dilated main pulmonary artery and its left and right branches, **(F)** volume-rendered (VR) imaging providing a visualization of the relationship between the dilated pulmonary artery, aorta, and trachea.

Electrocardiogram (ECG) was normal. Chest x-ray demonstrated normal lung markings and cardiac silhouette; however, the main pulmonary artery segment appeared prominently dilated. Given these findings and the need to assess for possible extracardiac vascular malformations, coronary computed tomography angiography (CCTA) was performed prior to echocardiography to obtain detailed three-dimensional visualization of the pulmonary artery and its branches. CCTA demonstrated significant dilation of the main pulmonary artery and its left and right branches (Figures 1A–1F) (4, 5).

**Figure 2:**
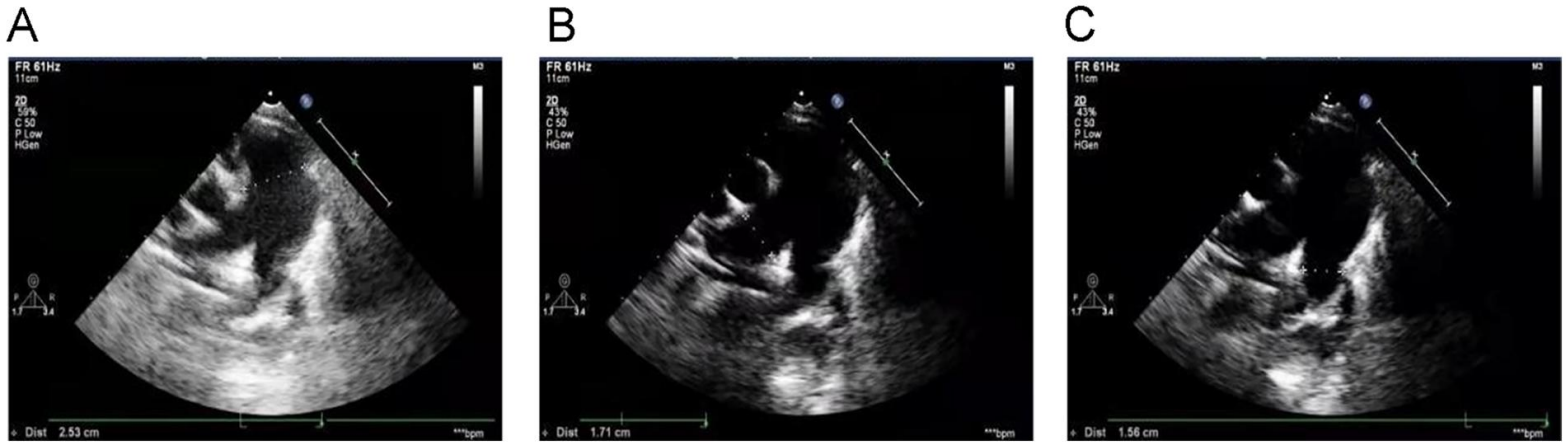
Echocardiography with TDI and EF measurement: **(A)** main pulmonary artery diameter (25 mm), **(B)** right pulmonary artery diameter (17 mm), **(C)** left pulmonary artery diameter (16 mm).

Echocardiography with tissue Doppler imaging (TDI) and ejection fraction (EF) measurement was subsequently performed and showed main pulmonary artery and branch dilation: main pulmonary artery diameter: 25 mm (*Z*-score: 5.32) (Figure 2A), right pulmonary artery diameter: 17 mm (*Z*-score: 6.08) (Figure 2B), left pulmonary artery diameter: 16 mm (*Z*-score: 5.3) (Figure 2C). The systolic pulmonary artery flow velocity was 1.1 m/s. During diastole, a small amount of regurgitation was observed, with a peak regurgitation velocity of 2.36 m/s and a pressure gradient of 22 mmHg.

No intra-cardiac or extra-cardiac shunts were detected on imaging. There was no evidence of chronic cardiopulmonary or arterial vascular disease. The diagnosis of IDPA was established based on the imaging findings and by exclusion of other causes.

During hospitalization, the patient was observed and no specific treatment for IDPA was administered. The patient was advised to undergo regular follow-up after discharge. In August 2021, the patient underwent a total splenectomy for management of hypersplenism. In March 2024, she was initiated on Bosentan (32 mg twice daily) and Hydroxyurea (0.25 g twice daily) due to hematologic indications. At the time of writing, the patient's treatment regimen remained unchanged, and the most recent follow-up echocardiogram performed in May 2024 showed a main pulmonary artery diameter of 25 mm (*Z*-score: 3.66), with no significant interval change.

## Discussion

### Pathogenesis and aetiology

The pathogenesis of IDPA remains incompletely understood. Assmann first suggested that abnormal differentiation of the arterial trunk during embryonic development could give rise to pulmonary artery dilation (3). A subsequently favored theory posits that defective development of elastic tissue within the pulmonary artery wall results in a focal weakness, predisposing the vessel to dilatation over time in response to normal hemodynamic forces (6). Support for this concept comes from case reports describing patients with concomitant hypoplasia or dilatation of the ascending aorta, which may indicate a generalized defect in arterial wall structure (7). While IDPA is often considered a congenital disease, it can also arise in association with other conditions, such as connective tissue diseases and genetic syndromes, highlighting the need for a thorough systemic evaluation in pediatric patients. Despite the enlargement of the pulmonary artery in IDPA, the vessel generally retains some elasticity, and patients typically demonstrate normal right ventricular and pulmonary pressures, with no significant chamber enlargement or wall thickening.

In the literature, most IDPA cases have been diagnosed in adulthood, although subtle or non-specific symptoms, such as mild dyspnea or cough, often date back to childhood but remain uninvestigated due to their benign nature (Table 1). Notably, Yang et al. (8) reported an antenatal diagnosis of IDPA, supporting the view that congenital factors are central to its etiology. However, acquired or secondary causes, including connective tissue disorders, vasculitides, and chronic pulmonary or cardiac conditions, must also be considered, especially in children with additional risk factors.

**Table 1:** Review of recent IDPA case reports published in the literature.

Ref #	Presenting complaint	Age at diagnosis	Gender	Imaging findings	Outcome/managment
Sueta et al. (10)	Progressive dyspnoea	86	F	Diagnosed on autopsy after admission and investigation for severe non-responsive congestive cardiac faliure	Deceased
Yang et al. (8)	Antenatal diagnosis	34 weeks	-	Doppler sonography: dialated PA, later enlarged to aneurysm at 37 weeks	Follow-up, no intervention
Betkier-Lipińska et al. (7)	Asymptomatic incidental murmur	18	M	Chest XR tomography: left pulmonary artery enlargement, CT: MPA dilatation and aneurysm, Echocardiogram: pulmonary artery dilatation	Follow-up, no intervention
Sharma et al. (3)	Progressive dysnpnoea	68	F	Chest XR: mild cardiomegaly of the right ventricular (RV) type with mild right atrial dilatation and massive enlargement of the main PA, Echocardiogram: NAD, CTPA: dilated main PA and left trunk, Cardiac catheterization: massively dilated the main PA.	Unknown
Kotwica et al. (11)	Asymptomatic incidental murmur	56	F	Chest XR- hilar mass, Echocardiogram: dilated left atrium, PA aneurysm, CT: left PA aneurysm	Follow up no intervention
Ugolini et al. (21)	Asymptomatic incidental murmur	16	F	Chest XR, left hilar mass effect, Echocardiogram: PA dilatation, MRI: MPA severely expanded, Right heart catheterization: normal PA pressure	Unknown
	Longterm exertional dynspnoea	47	F	Diagnosed on angiography and was under follow-up	Developed MPA aneurysm at 60 years old and had surgical management
	Asymptomatic incidental hilar mass	17	M	Echocardiogram: pulmonary artery dilatation, MRI: dilatation of MPA, Conventional angiography did not provide any additional information	Unknown

### Diagnostic criteria

The classic criteria described by Greene et al. (1949) (3) for IDPA diagnosis are: (1) Simple dilatation of the main pulmonary artery (diameter >30 mm) with or without branch involvement, (2) Absence of abnormal intracardiac or extracardiac shunts, (3) Absence of chronic cardiopulmonary disease, (4) Absence of arterial vascular disease (e.g., syphilitic arteritis, atherosclerosis affecting the pulmonary artery).

Deshmukh et al. (1960) (1) added a fifth criterion: normal right ventricular and pulmonary artery pressures, defined as a pressure gradient <10 mmHg at rest and <15 mmHg on exertion. In pediatric practice, the interpretation of pulmonary artery diameter relies on z-scores, which account for age and body surface area, and a *z*-score greater than +2 is generally considered abnormal (9). Thus, pediatric-specific reference values are essential for accurate diagnosis and risk stratification.

### Clinical manifestations

Clinically, most patients with IDPA are asymptomatic (1), and the condition is often detected incidentally during imaging for unrelated complaints. When symptoms do occur, they are typically mild and include exertional dyspnea, chest tightness, or cough. Some patients may develop progressive right heart failure or cyanosis if the condition remains undetected for many years (Table 1), as highlighted by autopsy findings in rare cases (10). Hemoptysis, although uncommon, is the most concerning presentation, as it may herald dissection or rupture of the pulmonary artery—a potentially life-threatening event (11). However, available evidence indicates that such complications are extremely rare in children, and the overall prognosis for pediatric IDPA is favorable.

### Differential diagnosis

IDPA is considered a diagnosis of exclusion and primarily requires differentiation from diseases that can cause secondary pulmonary artery dilation (1). Two prominent mimics are pulmonary valve stenosis and pulmonary artery aneurysms.

### Pulmonary valve stenosis

The diagnosis of IDPA is primarily one of exclusion, and it is essential to distinguish it from other causes of pulmonary artery dilatation. Two important mimics are pulmonary valve stenosis and pulmonary artery aneurysms. Pulmonary valve stenosis can cause post-stenotic dilation of the pulmonary artery due to turbulent flow, with the dilation often taking on a sac-like or spindle-shaped appearance (12). Historically, it was thought that right ventricular pressure would be significantly elevated in these cases, but recent data show that right ventricular pressure can be normal, and even giant pulmonary artery dilatation can occur in asymptomatic patients—sometimes unrelated to the severity of stenosis (13). Furthermore, patients with pulmonary regurgitation may also exhibit right ventricular and atrial enlargement, as was observed in our patient. This underscores the need for a comprehensive assessment, as the presence of pulmonary regurgitation may confound the clinical picture. Compared to IDPA, post-stenotic dilation from valve disease is more likely to produce compressive symptoms and right heart enlargement, and may be associated with distinct radiographic and electrocardiographic findings (12).

### Pulmonary artery aneurysm

Pulmonary artery aneurysms (PAAs) are typically defined in the literature as a marked dilatation of the pulmonary artery, most commonly with a diameter exceeding 40 mm (14–16). Importantly, even in patients with IDPA, an aneurysm may develop as part of a pathological continuum, as recognized in recent reports (14). PAAs, in contrast to IDPA, are usually focal and associated with underlying conditions such as pulmonary hypertension, infections, or congenital/acquired heart disease (17). Imaging, particularly CT angiography, is crucial for differentiating these entities and assessing the risk of complications. The distinction between IDPA and PAA is clinically relevant, as aneurysmal transformation may occur over time, and both entities may share overlapping risk factors or etiologies (14–16). In children, additional causes of pulmonary artery dilatation include congenital heart defects with shunt lesions (e.g., atrial or ventricular septal defect, patent ductus arteriosus), vasculitides (e.g., Kawasaki disease), and genetic syndromes with connective tissue involvement (18).

### Imaging

Chest x-ray may reveal a prominent pulmonary artery segment or hilar mass. However, findings are non-specific and must be interpreted in context.

Imaging plays a central role in the diagnosis and monitoring of IDPA. Chest x-ray often provides the first clue, revealing a prominent pulmonary artery segment or hilar mass adjacent to the left mediastinum (Table 1) (1). Echocardiography remains the initial imaging modality of choice in pediatric patients, allowing for assessment of pulmonary artery diameter, cardiac anatomy, and hemodynamics. It is non-invasive, readily available, and useful for both diagnosis and follow-up. However, echocardiography may have limitations in visualizing distal pulmonary arteries and their relationships to other thoracic structures, especially in cases where complex vascular anomalies or airway compression are suspected (19). In these scenarios, CT angiography can provide detailed three-dimensional visualization of the main and branch pulmonary arteries, as well as their anatomical relationships with the trachea, bronchi, and mediastinal structures (20). This comprehensive anatomical overview is particularly valuable for ruling out secondary causes of dilatation and for pre-surgical planning. Cardiac MRI offers the advantage of radiation-free imaging and allows for precise quantification of pulmonary artery size and flow, including assessment of pulmonary regurgitation and stenosis through advanced 4D flow techniques (19). The main limitation in young children is the frequent need for sedation or anesthesia, which can present logistical and safety challenges. Right heart catheterization and pulmonary angiography are now rarely required for diagnosis, but may be indicated in selected cases where non-invasive imaging is inconclusive or when invasive hemodynamic assessment is necessary.

### Treatment

There are currently no established guidelines for the management of IDPA, especially in children. Most patients—particularly those who are asymptomatic and have stable arterial dimensions—are managed conservatively with regular imaging surveillance. Surgical intervention is typically reserved for patients at high risk of rupture, those with symptoms attributable to compression of adjacent structures, or progressive dilatation. Importantly, pediatric intervention thresholds should be based on z-scores and clinical judgment, not adult cut-offs. Cases of giant dilatation in children have been reported without rupture or need for surgery; thus, the risk of rupture appears to be very low in the pediatric population (1).

### Specifics in this case

The initiation of Bosentan and Hydroxyurea in this patient was not specifically for the treatment of IDPA, rather, it was informed by the presence of comorbid hematological abnormalities, including hypersplenism and thrombocytopenia, as well as concerns regarding the potential development of pulmonary vascular disease or hemolytic complications. Bosentan, an endothelin receptor antagonist, is indicated for the management of pulmonary arterial hypertension, while hydroxyurea is utilized in the treatment of select hematologic disorders. In this patient, treatment was initiated as a precaution given the evolving clinical scenario and multidisciplinary consensus, although there was no direct evidence of pulmonary hypertension on hemodynamics. The rationale for splenectomy was management of hypersplenism and refractory cytopenias.

## Conclusion

IDPA is a rare and primarily congenital disorder, but secondary forms (including those associated with genetic and connective tissue disorders) must be considered, especially in children. Diagnosis relies on exclusion of other causes and careful imaging evaluation, with pediatric-specific criteria and z-scores being essential. While giant dilatation can occur, especially in pediatric patients, life-threatening complications such as rupture are exceedingly rare. Most children with IDPA can be safely managed with surveillance and conservative measures.

## Data Availability

The original contributions presented in the study are included in the article/Supplementary Material, further inquiries can be directed to the corresponding author/s.
